# Research Progress of ECG Monitoring Equipment and Algorithms Based on Polymer Materials

**DOI:** 10.3390/mi12111282

**Published:** 2021-10-20

**Authors:** Lvheng Zhang, Jihong Liu

**Affiliations:** College of Information Science and Engineering, Northeastern University, Shenyang 110819, China; 2070910@stu.neu.edu.cn

**Keywords:** arrhythmias, polymer materials, deep learning, electrocardiogram, generative adversarial networks, myocardial ischemia

## Abstract

Heart diseases such as myocardial ischemia (MI) are the main causes of human death. The prediction of MI and arrhythmia is an effective method for the early detection, diagnosis, and treatment of heart disease. For the rapid detection of arrhythmia and myocardial ischemia, the electrocardiogram (ECG) is widely used in clinical diagnosis, and its detection equipment and algorithm are constantly optimized. This paper introduces the current progress of portable ECG monitoring equipment, including the use of polymer material sensors and the use of deep learning algorithms. First, it introduces the latest portable ECG monitoring equipment and the polymer material sensor it uses and then focuses on reviewing the progress of detection algorithms. We mainly introduce the basic structure of existing deep learning methods and enumerate the internationally recognized ECG datasets. This paper outlines the deep learning algorithms used for ECG diagnosis, compares the prediction results of different classifiers, and summarizes two existing problems of ECG detection technology: imbalance of categories and high computational overhead. Finally, we put forward the development direction of using generative adversarial networks (GAN) to improve the quality of the ECG database and lightweight ECG diagnosis algorithm to adapt to portable ECG monitoring equipment.

## 1. Introduction

### 1.1. Background and Motivation

Ischemic heart disease (IHD) causes over 8 million deaths globally every year [1], making it the leading cause of death. It contains different types of arrhythmias including atrial fibrillation, ventricular tachycardia, and in severe cases, myocardial ischemia (MI).

To identify and diagnose heart diseases such as myocardial ischemia caused by coronary arteries, it is usually necessary to obtain cardiac physiological information by analyzing heart deformation or heart rhythm. To this, the main methods for detecting IHD include an electrocardiogram (ECG) and elevated levels of troponin [1]. Among them, ECG is widely used in the clinical screening of MI and arrhythmia for its non-invasive, real-time detection results and relatively low cost. In the past, the identification and diagnosis of ECG always depended on the manual work of doctors, which consumed a lot of medical resources. With the development of computer technology, computer-aided diagnosis technology is widely used in the medical field [2,3,4], which can liberate valuable medical resources. These methods in the direction of ECG diagnosis include threshold-based methods, machine learning methods, and recently rapidly developed deep learning methods.

**Figure 1:**
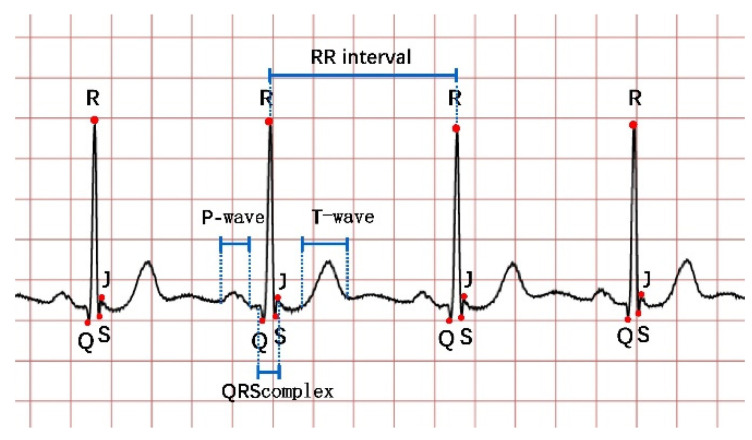
Schematic diagram of the electrocardiogram.

As the main method of heart disease screening, ECG signals obtained by connecting electrodes to the skin around the heart, which is generally presented in the form of multi-leads and multi-heart beats, contain rich physiological features. Twelve-lead ECG is the main screening tool for MI and arrhythmia. In clinics, the changes of waveform features in ECG are often used for judging different heart rhythms. Patients are divided into different types of arrhythmias or myocardial ischemia by the relative features, such as ST-elevation and depression or the shape of P wave, T wave, or the QRS complex [5,6,7,8]. According to the features of ECG, MI can be divided into several subspecies [5], and heart rhythms can be divided into five subspecies [9]. These studies show that electrocardiograms can quickly screen the myocardial health status of patients in the clinic. A typical ECG segment is shown in Figure 1.

With the development of sensor technology using flexible electrodes as materials and the development of embedded devices, ECG monitoring equipment continues to develop toward miniaturization and portability. Since 2020, the severe global situation of the COVID-19 epidemic has further strengthened the research and development needs of home portable medical equipment. Many ECG monitoring devices using new polymer materials have emerged, which surpass previous testing equipment in terms of detection accuracy, comfort, and convenience. ECG monitoring equipment that can be used at home also allows patients to shorten the time to find symptoms and improve the efficiency of diagnosis and treatment.

In addition to higher requirements for sensors and monitoring instruments, ECG monitoring equipment also needs accurate real-time detection algorithms to complete the fast and accurate processing of the collected signals. Deep learning, as a computer-aided recognition technology, uses the advantages of convolution neural networks and multi-layer perceptron to extract abstract physiological features from ECG signals, and it can identify diseases automatically by multi-layer nonlinear operation. The deep learning method has made many achievements in the analysis of medical data, such as disease classification and diagnosis [10,11], lesion location detection [12], organ edge detection [13], etc. This technology can liberate humans from mental work involving a large amount of image recognition and discrimination, thus allowing limited resources to be invested in more urgent work.

Meanwhile, the existing deep learning methods rely on large-scale data-driven techniques, which often have high requirements for the quality and quantity of data and the ability of the processing equipment. This makes the progress of diagnosis by the deep learning method move slowly in some scenarios. On the one hand, it is difficult to collect data from some special patients, or the amount of data is seriously unbalanced. On the other hand, in some atypical applications, such as home or travel, the use of deep learning methods for health detection or diagnosis is difficult to achieve. These problems have become hot research fields.

This article briefly reviews the current development status of composite material sensors used in ECG monitoring, enumerates the research progress of portable ECG monitoring equipment, introduces the latest algorithms applied in ECG detection, and lists deep learning technologies and their performance. It also proposes improved methods and prospects for the insufficient data quality problems of the current deep learning methods and the difficulty of monitoring the ECG in atypical scenarios.

### 1.2. Contributions

The contributions to this area of research can be summarized as follows:

- Review the progress of portable ECG monitoring equipment and its polymer material sensors.
- Review the deep learning models applied to ECG and introduce their structures and characteristics.
- Review the studies on the identification of heart rhythm or MI using deep learning methods; point out their common problems.
- Introduce the available ECG databases and evaluation indexes that are used to evaluate deep learning methods.
- Put forward some suggestions on the methods of data enhancement, which can improve the performance of deep learning method models.
- Put forward the suggestion of a lightweight network structure, which is helpful to apply diagnostic equipment to atypical scenarios.

### 1.3. Paper Organization

This article is organized as follows. Section 1 describes the development of ECG monitoring devices and computer-aided diagnostic methods. Section 2 reviews the current research on portable ECG monitoring equipment and introduces the progress of polymer material sensors for monitoring equipment. Section 3 reviews the databases of ECG and the evaluation index of diagnostic methods, introducing the deep learning methods of ECG diagnosis in detail. Section 4 introduces the shortcomings of deep learning methods and the current development. Section 5 summarizes the current sensor development direction of ECG monitoring equipment and the current algorithm research direction.

## 2. Portable Monitoring Equipment and Sensors

### 2.1. Portable ECG Monitoring Equipment

The gold standard for ECG detection is to use a standard 12-lead ECG recorder to continuously monitor Holter for 24 or 48 h [14]. Such a system has authoritative diagnostic data and comprehensive monitoring effects but has strict requirements for the detection environment and high time cost, which is not conducive to popularization in complex application scenarios. In contrast, portable ECG monitoring equipment can enter many complex life scenes in a more portable and comfortable way at the cost of acceptable accuracy loss, and it can acquire ECG detection data in a more flexible way. According to statistics, this can greatly shorten the time required to start diagnosis and treatment, reduce unnecessary referral or hospitalization costs [14], and win valuable treatment time for patients. When the world is under pressure from the spread of new coronavirus pneumonia, portable ECG monitoring equipment that can be used at home becomes more and more necessary.

**Figure 2:**
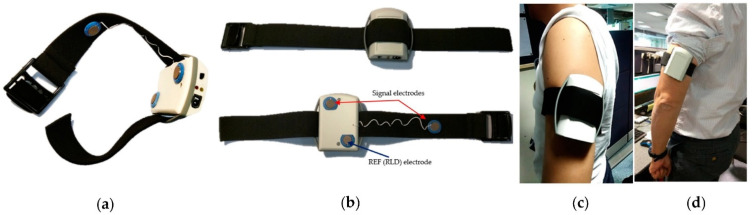
(**a**,**b**) show the electrode arrangement and the appearance of the wearable device; (**c**,**d**) show the scene of a person wearing an arm-type wearable ECG monitoring device [20].

There are many types of portable ECG monitoring equipment in wearable form. Lee et al. [15] used sensors implanted under the skin to monitor ECG signals and uploaded the collected data to handheld terminals and hospitals via wireless networks. The signal monitored by the subcutaneous sensor has high accuracy but at the expense of convenience. In contrast, many studies choose a non-invasive way to obtain ECG signals, and its sensor carriers include smartwatches, sports shirts or T-shirts, and other forms to meet the monitoring needs in complex scenarios [16]. Beach et al. [17] proposed using a wristband-type ECG sensor to collect data and upload the data to a data center for analysis. Zhu et al. [18] introduced devices that can be worn by pregnant women that can collect the fetal heart rate and transmit it to the hospital for remote monitoring. Choi et al. [19] designed a device that measures the driver’s ECG signal through a capacitive sensor in a non-contact situation so that high-risk groups in special environments can effectively monitor their health. A typical portable and wearable arm-type ECG monitoring device is shown in Figure 2 [20].

### 2.2. Polymer Material Sensor

**Figure 3:**
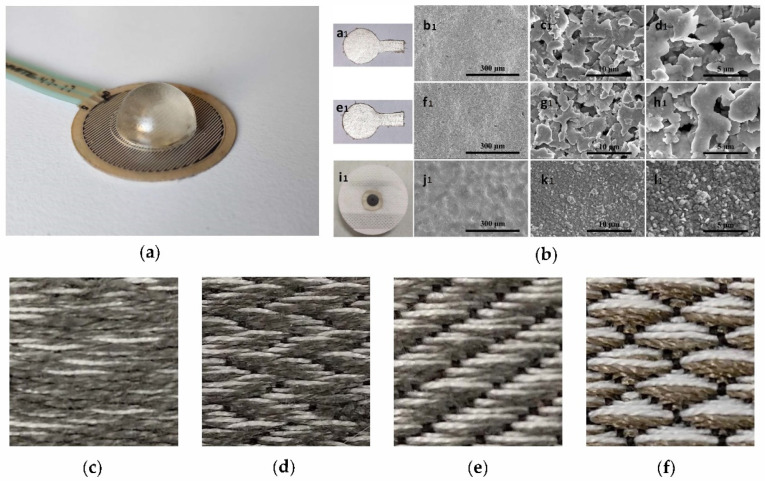
Monitoring sensors of different materials: (**a**) A kind of force-cardiography sensor [26]; (**b**) from a1 to l1, there are three kinds of wearable flexible silver electrodes, including an electrode based on the top grain layer (**a1**–**d1**), split layer (**e1**–**h1**), and the standard Ag/AgCl electrode (**i1**–**l1**) [27]; (**c**–**f**) show the different weave structures of woven textile electrodes [28].

Sensors are an important part of portable ECG monitoring equipment. The quality of signal collection and wearing comfort are largely determined by the sensors. Flexible polymer material sensors have made great progress in these aspects. The typical sticky heart monitor patch is easy to use, comfortable to wear, and waterproof [21], but this kind of wet electrode sensor is troublesome to operate; the P wave signal quality is not good. The dry electrode can be quickly assembled and cleaned but is unstable and easy Motion artifacts appear. The semi-dry electrode can overcome the above shortcomings, but the release of the electrolyte is uncontrollable, resulting in signal instability [16]. Flexible electrodes have made great progress in recent years and have become a good substitute. The use of metal thin films and graphene thin films can be used to make flexible dry electrodes. By using silicon rubber and special carbon materials, the expansion capacity and elasticity of these electrodes are often higher than that of metal electrodes [22]. Fabric electrodes and conductive polymer electrodes are also increasingly being used as sensors for wearable devices. Zhang et al. [23] confirmed that their polymer composite electrodes are as effective as Ag/AgCl electrodes in measuring ECG signals and have excellent scalability. In the direction of non-contact sensors and implantable sensors, flexible electrodes using composite materials have achieved new breakthroughs in signal quality [24,25]. Figure 3 shows the shape of the ECG sensor of different types of materials [26,27,28].

The new composite sensor greatly improves the convenience, environmental adaptability, and economy of the sensor, making the miniaturization of ECG monitoring equipment possible. In the future, these devices will become part of the Internet of Things, developing in the direction of small size, long battery life, convenience, and fashion, making the collection of ECG signals easier. Sensors made of other materials, such as fiber optic sensors, are currently emerging in respiratory monitoring. In the future, ECG monitoring sensors with smaller volumes and higher sensitivity may be based on fiber optic technology. At the same time, the massive amount of data generated urgently need to be processed more efficiently, and the application of intelligent algorithms is indispensable.

## 3. Deep Learning Methods and Evaluation

The ECG detection algorithm is the software core of the portable ECG monitoring device, which enables users to detect abnormal health conditions and trigger alarms in time. In this paper, the methods of computer-aided ECG detection are divided into two categories: one includes the traditional detection methods, including mathematical algorithms and machine learning methods that require manual feature extraction, and the other includes end-to-end deep learning methods.

### 3.1. ECG Diagnosis Algorithm

#### 3.1.1. Traditional Methods

**Figure 4:**
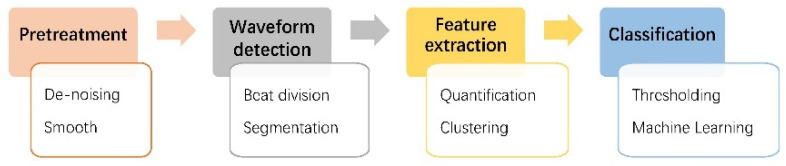
Traditional computer-aided methods for the diagnosis of ECG.

Early computer-aided programs often use manually extracted morphological features to compare the threshold [29,30], or the morphological features extracted manually are classified by the machine learning method [10,31,32,33,34,35]. The diagnosis process can usually be divided into four steps [1], followed by pretreatment, waveform detection, feature extraction, and classification, of which the most critical step is feature extraction. The detailed steps are shown in Figure 4. The reliability of these methods depends on the accuracy of features extraction, so they have poor adaptability to different datasets with morphological features in different environments and lead to the poor portability of the methods. In addition, the feature extraction requires a high level of ECG theory of the algorithm designer, so it is easy to introduce additional errors into the algorithm.

#### 3.1.2. Deep Learning Methods

With the continuous strengthening of the ability of data storage and the continuous improvement of computing power, deep learning algorithms have made breakthroughs in many fields. Especially in the field of medical imaging, the use of deep learning methods includes disease diagnosis of magnetic resonance imaging [36,37], diagnosis and reconstruction of computed tomography [38,39], and the rhythm and MI diagnosis of ECG [6,40,41,42,43,44,45].

A deep learning algorithm learns the features of the input signal independently through the multi-layer convolution neural network, which is usually the end-to-end discrimination way from the original information to the classification result. This method not only avoids the step of manual feature extraction but also can obtain more comprehensive and hidden physiological information by extracting the features of different dimensions in the original data. Therefore, the ECG detection algorithm using the deep learning method greatly improves the accuracy of the computer-aided algorithms in heart rhythm classification and MI discrimination. It has been proved that the deep learning algorithm model can be classified and judged with higher accuracy than that of professional doctors after appropriate data training [46].

As a data-driven research method, the application and evaluation of deep learning methods cannot be separated from high-quality ECG data. Some commonly used ECG databases and performance evaluation indicators of deep learning methods will be introduced firstly.

### 3.2. Database

Most of the ECG databases suitable for deep learning methods are collected from clinical cases and have rich physiological features. At present, the authoritative ECG databases in the world are mainly the MIT-BIHA ECG database of Massachusetts Institute of Technology [47], the MIT-BIHSTC database [48], the MIT-BIHNST database [49], the AHA ECG database of the American Heart Association, the PTB ECG database provided by the German National Metrology Agency [50], and so on. Their main information is shown in Table 1.

In addition, there is the EU’s CES ECG database, which contains 1000 short-term ECG records with 12 or 15 leads, and the EU’s ST-T database contains multiple 2 h data with a sampling frequency of 250 Hz, which can be downloaded free of charge or paid for on its website.

However, due to the large difference in the number of diagnosed patients with different types of diseases, the data collected from these patients have the problem of a small number of samples or imbalance in the number of samples. This will make the deep learning model tend to judge the heart rhythm type as the one with a larger number in the training set, which will have a negative impact on the accuracy of the model and should be avoided in the training stage of the deep learning model. The specific solutions are described in detail in Section 4.

### 3.3. Evaluation Index

In many proposed studies, the following indicators are usually used to evaluate the performance of deep learning models: accuracy (*ACC*), sensitivity (*SEN*), specificity (*SPE*), and sometimes precision (*PRE*) [8,51,52,53]. Accuracy indicates the percentage of correct prediction in the total; sensitivity indicates the proportion of all positive cases that are correctly classified, reflecting the classifier’s ability to identify positive cases; specificity indicates the proportion of all counterexamples that are correctly classified and reflects the classifier’s ability to identify counterexamples; precision indicates the proportion of positive examples that are positive examples. The definitions of accuracy, sensitivity, specificity, and precision are shown as follows:

(1) $$ACC=\frac{TN+TP}{TN+TP+FN+FP}\times 100\%$$

(2) $$SEN=\frac{TP}{TP+FN}\times 100\%$$

(3) $$SPE=\frac{TN}{TN+FP}\times 100\%$$

(4) $$PRE=\frac{TP}{TP+FP}\times 100\%$$

where TP is the positive sample that is judged correctly, TN is the negative sample that is judged correctly, FP is the positive sample that is judged wrong, and FN is the negative sample that is judged wrong. In the deep learning model of heart rhythm judgment, the evaluation index of each type of heart rhythm is usually counted separately, and then, the arithmetic average of all kinds of heart rhythm evaluation indexes is carried out to get the overall evaluation index data of the classifier.

The confusion matrix is also often used in the study of rhythm recognition [6,41,43,46]. It is a matrix in which all rows are class labels, each column represents the predicted category, and each row represents the true attribution category of the data. Its definition is shown in Table 2. The overall accuracy of the algorithm can be directly reflected by using the confusion matrix.

### 3.4. Method Review

The deep learning method using a convolution neural network brings two major characteristics from its structure. First, its convolution structure can independently learn parameters through a backpropagation mechanism to extract the features of ECG signals.

**Figure 5:**
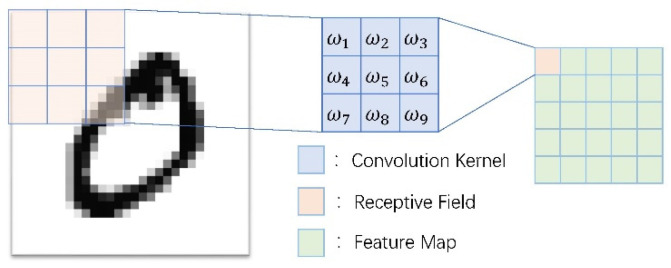
Convolution process of image.

A typical two-dimensional image convolution process is shown in Figure 5. At the same time, the multi-layer neural network enables the convolution structure to learn high-level abstract semantic features; this structure enables the deep learning network to identify the physiological ECG semantic features that are difficult for the human eye to perceive, and it can achieve high accuracy in diagnosis and discrimination.

At present, there are mainly two ways to deal with ECG signals. One is to convert ECG signals into digital one-dimensional sequences and change the deep learning model to make it able to process one-dimensional signals. Another method can deal with old ECG images that are difficult to digitize [54] or transform digital ECG sequences into two-dimensional image data [55]; then, it can use a typical deep learning model for image processing.

#### 3.4.1. LeNet

**Figure 6:**
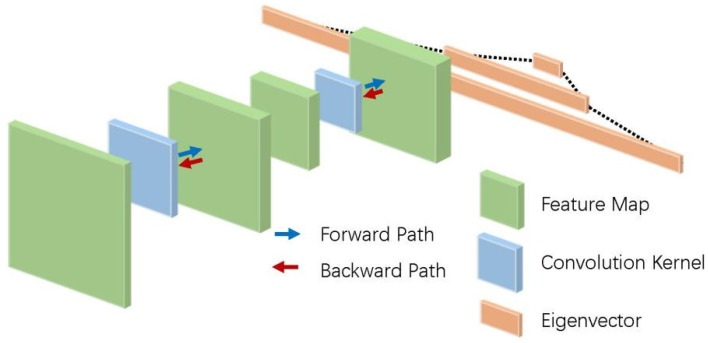
Structure of LeNet-5.

Since the convolution neural network structure with five layers was proposed by LeCun et al. [56], the structure of the deep learning network has been updated continuously. With the deepening of the number of layers and channels of the network and the increasing number of model parameters, the learning ability and generalization ability of the model to complex data have been greatly improved. Figure 6 is a typical structure of LeNet-5. Many effective deep learning methods are developed from this simple structure.

#### 3.4.2. AlexNet

Krizhevsky et al. [57] proposed the AlexNet structure to improve the performance of the deep neural network; the network structure is similar to LeNet but deepened to eight layers, and the activation function is changed from Sigmoid to ReLU. These changes make the model parameters larger, so the generalization ability is stronger, and the test on the ImageNet data set gets the highest score at that time. Many methods similar to the AlexNet structure or its variants have been applied to the discrimination of ECG [6,43], including the use of multi-port inputs to improve the model’s ability to extract features from different dimensions of ECG [41,58].

#### 3.4.3. ResNet

**Figure 7:**
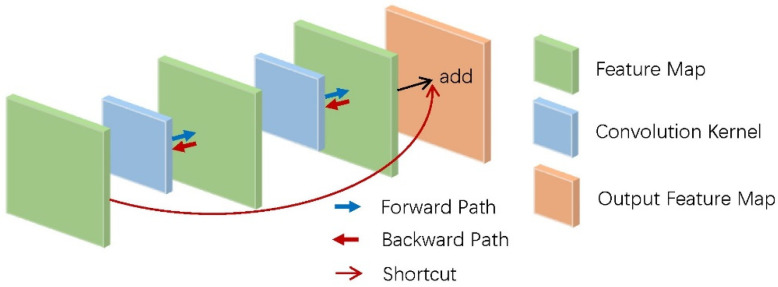
Structure of a basic residual block.

Theoretically, models with deeper layers of neural networks can achieve better feature extraction results on data sets, but when the depth of the network reaches a certain number, many studies have found that the training of the network becomes unstable and the effect of classification becomes worse—that is, there is the problem of network degradation, which is often caused by the cumulative changes of network parameters such as gradient disappearance or gradient explosion [59,60]. To solve this problem, the residual network is composed of a residual block structure proposed by He et al. [61] that effectively transmits the input information backward and reduces the problem of network degradation. The residual block structure is shown in Figure 7. The network depth using the residual block structure can often reach hundreds or even thousands of layers, which makes this deep learning model able to achieve better fitting and generalization effects in large datasets.

**Figure 8:**
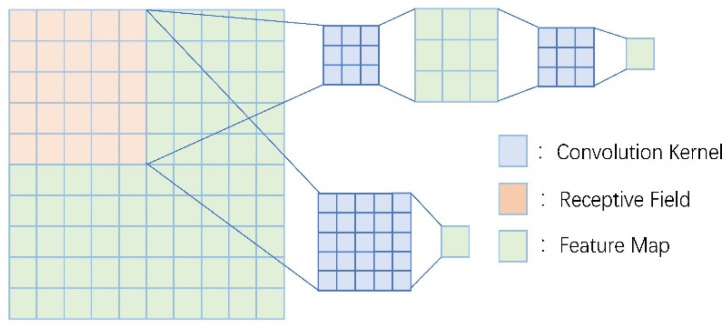
Compared with the large convolution kernel that requires 25 parameters, the small convolution kernel has the advantages of fewer parameters (18 parameters) and deeper network depth under the same receptive field.

In addition, the convolution kernel with a size of 3 × 3 used by Resnet has more advantages than the large-size convolution kernel in AlexNet, and it needs fewer parameters to be calculated under the same receptive field, as shown in Figure 8.

#### 3.4.4. RNN

**Figure 9:**
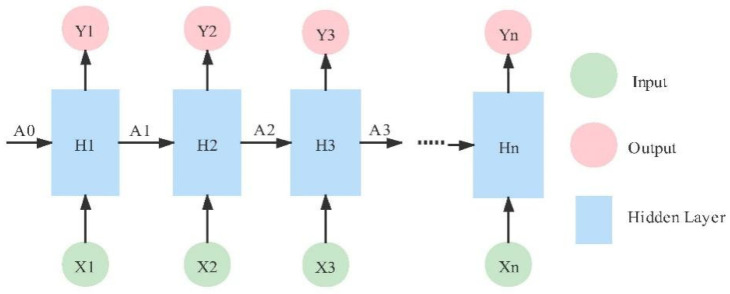
Typical structure of RNN.

The recurrent neural network (RNN) is originally suitable for time-related sequences such as speech and text and is composed of several horizontal hidden layers [62]. Its structure gives it the memory of the input sequence, as shown in Figure 9, so it has a certain advantage in learning the nonlinear features of the sequence. The digital signal contained in ECG can also be regarded as a time-related sequence. Some studies have applied RNN and its improved variant networks, such as LSTM and the GRU structure, to ECG detection, and they achieved certain results [45,63].

### 3.5. Evaluation

Many models of deep learning have been applied to the classified diagnosis of ECG, and these studies have improved the performance of ECG diagnosis to a new level. Compared with many traditional methods, the advantage of the deep learning method is that it does not need to extract features manually, simplifies the design process of the algorithm, and avoids additional errors, which is also called the end-to-end model. The detailed performance of some studies using deep learning methods to detect different rhythms or MI is shown in Table 3.

With the continuous improvement of the performance of the model, the reliability of deep learning technology as an auxiliary diagnosis method has been gradually recognized. The ResNet network with 34 layers of depth is proposed by Awni et al. [46] for heart rhythm classification on the ECG data obtained by the ECG acquisition equipment that has been put to use; the heart rhythm detection results have exceeded the expert doctors in accuracy. In the foreseeable future, with the update of the model structure and the improvement of training data, deep learning technology will be able to perform the task of ECG detection under more complex disease categories and broader detection conditions.

## 4. Challenges and Solutions

Using deep learning technology can achieve high accuracy in the classification of heart rhythm or the diagnosis of MI, but there are still some challenges until now, which can seriously affect the final diagnosis results of ECG.

- Class Imbalance

Since the number of patients with different heart disease is different in the clinic, when the current public ECG dataset collects ECG in the clinic, the amounts of different types of heart rhythm data are also different, which leads to serious class imbalance. To obtain the highest possible *ACC*, the model trained by the deep learning method tends to predict the new sample into a larger number of known categories. This will make it more difficult to detect heart rhythms that are rarely seen clinically, such as the fusion of pacing and normal beats. If class imbalance occurs, the *ACC* of diagnosis might be very high, but the *SEN* of the model will be significantly reduced. As can be shown in Table 3, models evaluated with common datasets generally perform poorly in terms of *SEN*, and it is clear that such results are unacceptable for the diagnosis of patients with rare diseases.

- High Computational Cost

The detection scene of ECG mainly exists in all kinds of hospitals, and the equipment specifications of ECG collection are different, which makes it difficult for patients to get timely and convenient ECG diagnoses. With the development of the Internet of Things (IoT), miniaturized medical devices will enter people’s homes, and portable ECG detection devices will be widely used in the future. This kind of equipment should have the characteristics required by IoT devices such as small size, low power consumption, and strong anti-interference ability; at the same time, as auxiliary diagnostic equipment, its *ACC* must be within an acceptable range. The deep learning algorithm is very suitable for this kind of automatic equipment due to its high *ACC* and automatic diagnosis characteristics, but the current popular deep learning model’s parameters often reach several megabytes to hundreds of megabytes, which have high requirements for equipment. It is difficult to be competent for the task of real-time computing in miniaturized IoT devices. Therefore, there is an increasing demand for a deep learning model with a simplified network structure, small parameter volume, and good performance.

### 4.1. Data Expansion by Generative Adversarial Networks

To solve the problem of class imbalance, a method of data enhancement is to rotate and cut the input image to increase the amount of data, which makes it easy to change the morphological structure of the original features and does not add new reference features, so the effect is not good. Another method is to oversample the categories with less data until its number is similar to the number of other classes. This method still cannot provide new reference features, and its effect is very limited [64].

The best way seems to be to get more new data of the same type. In the case that it is difficult to obtain more rare types of ECG signals in the clinic, the needed simulated ECG signals can be obtained by the mathematical modeling method [65,66]. This method can generate very standard beats, but the generated beats of the same type are standard and similar, and the process of generating different types of beats is also very complicated.

Compared with the mathematical modeling method, the generative adversarial networks (GAN) based on a deep learning backbone network can generate new signals with diversified features, which is more suitable for the expansion of ECG data. Making use of the characteristics of convolution neural network, the generator in GAN can effectively extract the abstract features of the input signal; using the skillfully set objective function Formula 5 [67], the generator and discriminator can improve the utilization ability of features in the continuous update confrontation and finally enable the generator to learn the data distribution of samples and generate new signals that can deceive the discriminator. The optimization process of the generator and the discriminator is shown in Equation (5).

(5) $$\underset{G}{\min}\;\underset{D}{\max}\;E_{x\sim {\mathbb{P}}_{r}}\left[\log\left(D\left(x\right)\right)\right]+E_{\tilde{x}\sim {\mathbb{P}}_{g}}\left[\log\left(1-D\left(\tilde{x}\right)\right)\right]$$

where x and $\tilde{x}$ represent the original images and the generated image; G and D represent the generator and discriminator, respectively. E is the distribution of data, ${\mathbb{P}}_{r}$ and ${\mathbb{P}}_{g}$ are the original distribution of data and distribution of the generator.

**Figure 10:**
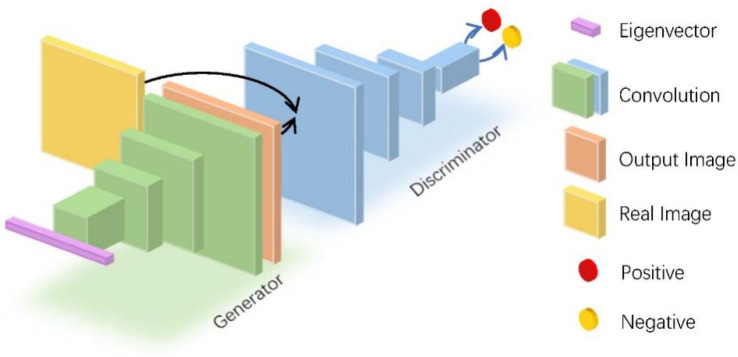
Typical structure of GAN of Image generated from eigenvectors.

The backbone of GAN consists of two neural networks, a generator and discriminator, and its typical structure is shown in Figure 10. Since GAN was proposed, there have been many improved structures [68]. In the field of computer vision, they have been applied in generation [69], restoration [70,71,72], style transfer, and natural language processing in text generation [73].

Some studies have begun to apply GAN to the generation of ECG signals [64,74,75]. They use one-dimensional ECG signals as training sets to train GAN to produce new data similar to real collected signals. The ECG signal generated by GAN has the same main features as the target category signal. On this basis, due to the change of random parameters during generation, the generated signals of the same type are not exactly the same, which is very similar to the difference between the real data. The expanded dataset generated by GAN solves the problem of class imbalance, and the performance of the deep learning model trained by it is further improved; also, the *SEN* of discriminating heart rhythm categories with a small number is significantly improved [74,75].

Using GAN can expand the existing unbalanced data, and its generation effect is better than that of many traditional methods. At the same time, the research on the generation of ECG data is mainly focused on the generation of a single heartbeat, and the way to improve the generation and quality of long heartbeat data is still a problem to be solved. The GAN network with improved structure and loss function seems to be a good way to solve this problem.

### 4.2. Lightweight Algorithm

**Figure 11:**
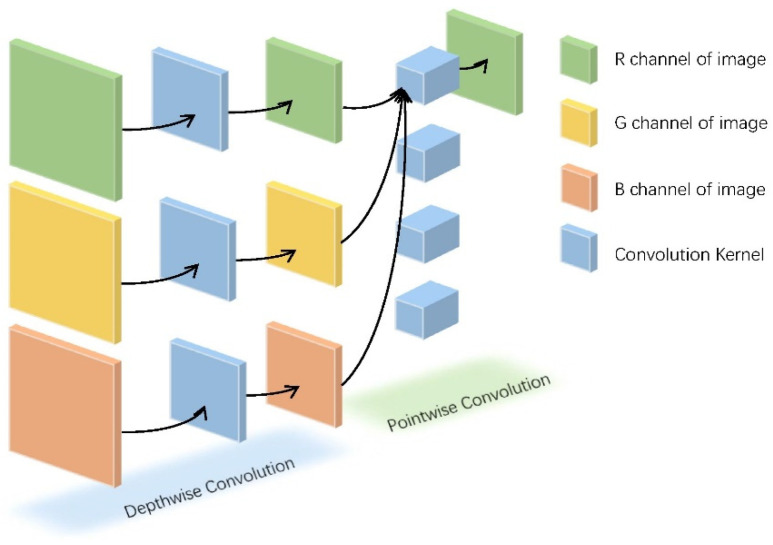
Depth-level separable convolution structure of MobileNet.

Today, with the vigorous development of embedded devices, the miniaturization of auxiliary medical devices has gradually spread to people’s homes. Since 2020, the severe global situation of COVID-19 has also given birth to many household portable medical devices. Many studies are devoted to miniaturizing ECG monitoring devices [76,77]. The mainstream deep learning algorithm models have high requirements for the number of parameters and equipment, so they are difficult to adapt to embedded devices. A miniaturized network structure MobileNet for embedded devices is proposed for use in this scenario [78]. The depth-level separable convolution structure in MobileNet can be regarded as composed of a two-part convolution of depthwise convolution and pointwise convolution, which are shown in Figure 11. When the performance of MobileNet on the ImageNet dataset is not much different from that of the VGG model, the number of parameters of MobileNet is much less than that of the VGG model, as shown in Table 4.

**Figure 12:**
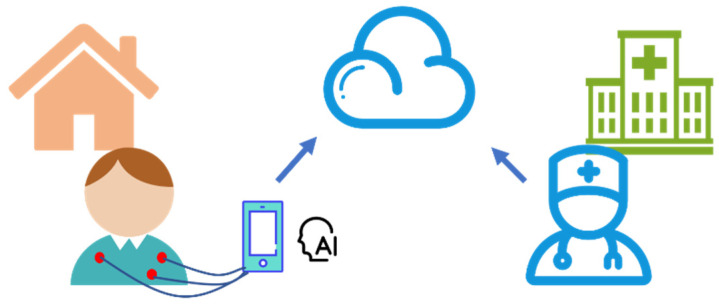
Application scenario of IoT for ECG diagnostics. Patients can diagnose locally through offline algorithms and share diagnostic information with hospitals through cloud services.

The portable ECG detection equipment used in studies uses MobileNet as the backbone network, which can obtain the user’s ECG in real time and has a high accuracy rate [79]. The system uses three-lead ECG monitoring equipment to collect ECG signals and uses the local system composed of raspberry pi and an Intel NCS2 coprocessor to embed algorithms for intelligent detection. When an abnormal heart rhythm is detected, the device connected to the wireless network will send an alarm signal and detected abnormal data to the cloud data center, and the hospital will provide timely assistance after obtaining the data. The conceived scenario is shown in Figure 12 [79]. This study verifies the practicability of MobileNet and confirms that the deep learning algorithm can run effectively on small embedded devices. The lightweight depth neural network will become a kind of important algorithm of IoT, providing accurate and fast diagnosis.

In the future, the research direction of the structure of the new network should not only make the number of layers deeper and larger but also pay attention to the efficiency of the network, so that the network with fewer parameters can meet the existing requirements as much as possible and can be popularized to more devices of the IoT.

## 5. Discussion and Conclusions

### 5.1. Discussion

Generally speaking, with the advancement of polymer flexible electrodes, there is still a lot of room for improvement in wearable ECG monitoring equipment, and a more comfortable non-invasive detection method will improve the patient’s experience while improving accuracy. The deep learning method is still insufficient in the field of ECG detection. In the face of a broad prospect of household medical equipment market, in fact, it needs to further solve the problems of class imbalance and huge parameters. The existing GAN can effectively expand the training data, but there are still some problems that the new data are not rich enough, and the long heartbeat is difficult to generate. The new GAN structure and improved objective function can be used to solve the existing problems. The existing small network can achieve a certain calculation accuracy when using less system overhead, but it still cannot achieve the latest deep learning network diagnosis effect. GAN still has great development potential in its own structure to generate samples closer to the real data distribution. Lightweight algorithms need to continue to improve performance in terms of compression parameters and improving accuracy.

### 5.2. Conclusions

In this paper, we first reviewed the development status of portable ECG monitoring equipment and its polymer material sensors. New materials have great potential for solving the problems of high power consumption and the low accuracy of monitoring equipment. The future development of ECG monitoring equipment features smaller, more comfortable, and lower-cost designs. Then, we introduced in detail the real-time detection algorithm used for ECG monitoring equipment, mainly the deep learning methods of heart rhythm classification and MI detection in ECG. The ECG databases and evaluation indicators used in deep learning methods are introduced, and the relations and application effects of different deep learning frameworks are reviewed in detail. At present, deep learning methods exceed the performance of traditional methods in most indicators and can be used as clinical assistance in some detection situations. However, in the face of multiple classification methods of different heart rhythms and complex collection environments, it is difficult to achieve stable and satisfactory sensitivity. This paper puts forward some suggestions for improvement from the aspects of an expanding dataset and lightweight network structure and gives the latest progress. These two algorithms seem to have application prospects in improving the performance of ECG monitoring equipment algorithms. Their network structure is still improving and will have better performance in the future.

## Figures and Tables

**Table 1:** Detail of ECG database.

Database	No. of Records	Duration (Per Record)	Sampling Rate	Leads	Classes
MIT-BIHA	48	30 min	360 Hz	2	17
MIT-BIHSTC	28	24 min	360 Hz	2	5
MIT-BIHNST	2	30 min	360 Hz	2	5
TELE	250	48 min	500 Hz	1	-
QT	105	15 min	250 Hz	2	-
AHA	155	180 min	250 Hz	2	8
PTB	549	2 min	1 kHz	16	13

**Table 2:** Confusion matrix.

	Prediction Label	1	2	3	4	5	Total
Real Label							
1		N11	N12	N13	N14	N15	N1X
2		N21	N23	N24	N25	N26	N2X
3		N31	N32	N33	N34	N35	N3X
4		N41	N42	N43	N44	N45	N4X
5		N51	N52	N53	N54	N55	N5X
total		NX1	NX2	NX3	NX4	NX5	-

**Table 3:** Performance of different ECG diagnosis approaches.

Author	Classifier	Database	Classes	*ACC*	*SEN*	*SPE*
Kiranyaz et al. [6]	CNN	MIT-BIHA	5	98.90%	95.90%	99.40%
Acharya et al. [43]	9 layers-CNN	MIT-BIHA	5	93.47%	96.01%	91.64%
Wang et al. [41]	Multi input CNN	MIT-BIHA	4	98.67%	86.39%	-
Chang et al. [63]	LSTM	CMUH	12	90.00%	-	-
Hasan et al. [58]	Multi input CNN	MIT-BIHA	4	97.70%	-	-
Chen et al. [45]	LSTM + CNN	MIT-BIHA	6	99.32%	97.75%	99.51%
Jafarian et al. [11]	ResNet	PTB	MI detection	98.21%	97.50%	98.01
Zhi Li et al. [42]	31 layers-ResNet	MIT-BIHA	5	99.38%	94.54%	-
She et al. [40]	ResNet	clinical 8-lead ECG signals	MI detection	99.75%	99.52%	99.81%

**Table 4:** Performance of different models on ImageNet.

Model	*ACC* on ImageNet	Multi-Adds (Million)	Parameters (Million)
1.0 MobileNet-224	70.60%	569	4.2
GoogleNet	69.80%	1550	6.8
VGG-16	71.50%	15,300	138
